# The Prince of Wales's Hospital Fund

**Published:** 1900-06-30

**Authors:** 


### The Institutional Workshop.

### THE PRINCE OF WALES'S HOSPITAL FUND.

We have received the following from the hon. secretaries of the Fund:—

Report of the Executive Committee of the Prince of Wales's Hospital Fund for London upon the Memorial to His Royal Highness the Prince of Wales, K.G., from the Council of the Metropolitan Radical Federation, dated March 17th, 1900.

The memorial (which incorporates an article in the *Contemporary Review* by the Hon. Stephen Coleridge), after stating that the Federation represents 40,000 to 50,000 members of Working Men's Radical Clubs and other Radical organisations in London, and that the mass of the population, by reason of the dread of entering infirmaries caused by the social ignominy that it incurs, and by the social and political disqualifications that accompany it, are absolutely dependent on the London hospitals for the skilful treatment of their more serious diseases and accidents, sets forth two matters of complaint, which are summarised as "one of the gravest scandals of our time." They are as follows:—

"1. A widely-spread idea that there is too much a tendency nowadays on the part of medical doctors and students attending the London hospitals to regard the patients that come under their hands as so much material for experiment, and the memorial in this connexion says: 'It seems to be an axiomatic truth that if experiments must be made on a living subject, the proper subject must be the person of the patient himself.' An inference is drawn that the popular suspicion is based on sufficient ground.

"2. That grants of money from the general receipts of hospitals are devoted to medical schools; that since the inauguration of this Fund such grants have been increased; that certain hospitals in London in receipt of large sums of money from the Prince of Wales's Hospital Fund allocate considerable grants of money from their general receipts, among which the contributions from the Prince of Wales's Fund figure, to the medical schools and to the medical training of the students attending the schools of those hospitals. And in the said article the writer, after referring to your Royal Highness's statement in 1897 that there was no intention of devoting any part of this Hospital Fund towards the support of the medical laboratories, says: 'Upon the committee of the Prince of Wales's Hospital Fund, therefore, there lies the heavy responsibility of disregarding His Royal Highness's clearly expressed beneficent intentions.'"

With regard to the first allegation the Executive Committee, under whose direction the hospitals have been recently subjected with their own accord to careful and expert inspection, are deliberately of opinion that it is unfounded in fact. No particulars are given which can be dealt with by evidence in detail. The charge is general, founded on an alleged "widely spread idea," and on what "seems to be an axiomatic truth." The committee from their knowledge of the hospitals doubt, and have reason to doubt, that any such idea is at all widely spread, and they are sure that, whether widely spread or not, it is erroneous and does grave injustice both to the medical doctors and to the students. The axiom appears to them questionable, and the inference drawn from it unjustifiable. Upon this loose and somewhat scandalous charge against a great profession, they do not consider they are called upon to say more.

The argument of the second allegation resolves itself into this. The Fund subscribes to a hospital, a complicated organisation, the main object of which is surgical and medical aid to the poor. The hospital comprises a medical school. Therefore, the Fund supports the medical school. The Executive Committee loyally adhered to your Royal Highness's statement in 1897 that there was no intention of devoting any part of the Fund towards the support of medical laboratories. The honorary secretary of the Metropolitan Radical Federation has already been informed by the Executive Committee of this Fund that no grant has been made from this Fund towards the support of medical laboratories, either in the case of the Middlesex or any other hospital, and that all hospitals applying are considered according to their needs and merits as institutions for the relief of the suffering poor. The utmost that can be made of the charge is that some minute unascertained portion of a grant for general purposes may have percolated under the administration of the hospital concerned into a medical school or laboratory. But even this has been guarded against; and, so far as the committee can ascertain, no such percolation has actually occurred.

The awards made by this Fund to the hospitals have been made with certain conditions, following as nearly as possible the recommendations of the visitors who have inspected and reported upon them, and these awards, and the objects for which they were made, are stated in the annual reports of the Fund, and have also been published in the public press. In no case can there be found in these reports an award given for a medical school or laboratory.

Nor can it be fairly said that the hospital authorities have, in fact, used any parts of such grants for medical schools and laboratories. It is alleged that certain payments in connection with the schools have been increased since this Fund was started, but even if this be so, it by no means follows that such increases were made in consequence of the help given by this Fund to the hospitals in question. In three cases (Middlesex, London, and St. Mary's) out of the four quoted, the receipts of these hospitals, excluding any grant from this Fund, were considerably over £40,000 more in 1897 than in 1896. These hospitals had, therefore, much greater resources out of which larger grants could have been made to the schools quite irrespective of any grants from this Fund. In the remaining case, that of Guy's Hospital, where £116 is mentioned as being paid for the school in 1896, and £595 in 1897, the hospital's receipts, including £8,000 from this Fund, were less than those of 1896—thus giving a result antagonistic to the theory of greater funds, greater help to the schools. There is no reason for supposing that Guy's, any more than the other three hospitals, used the contributions from this Fund, or any part of them, for the purposes of the medical school of the laboratory. It is to be observed that, accepting as correct the sum stated to have been for the benefit of the schools, they do not rise or fall in proportion to the grants from this Fund, and have, from this point of view, no corelation with such grants; indeed, it is quite clear that the amounts paid in connection with the schools, which bear only a very small proportion to the total annual expenditure of the hospitals, are determined by factors other than the receipts from the Fund. In none of the hospital accounts does it appear that any part of the grant from this Fund has been allocated to the schools. In the case of the Middlesex Hospital, which is given special prominence in the article, £600 was disbursed in 1897 in connection with the school, forming part of a total annual expenditure of £30,000. The grant from this Fund in 1897 is quoted in the article as £1,000, but, as a matter of fact, the amount was £2,925. Looking at the varied items of receipts, it is not fair and reasonable to infer that because money from this Fund is included in such receipts any part of it must necessarily have been used for the school, especially when it is shown that among the other receipts there are annual dividends of £6,615, of which £2,076 are for the Cancer Fund. It would be more fair and reasonable to assume that a portion of such dividends is properly available for school purposes, and this presumption is borne out by a recent statement in the public press by Sir Ralph Thompson, who refers to some bequests to this hospital which are given not only for the cure and care of patients suffering from cancer, but also for the purpose of investigating and promoting a general knowledge of treating it.

In administering this Fund, the committee have endeavoured in all cases to aid the hospitals in the manner best suited in the case of each hospital to benefit the sick and suffering poor of London, and they have no reason to believe that the eminent authorities who are associated with the large and important hospitals that have medical schools have done otherwise than act to the best of their ability in promoting the highest interests of the hospital patients.

